# The intriguing relationship between the ABO blood group, cardiovascular disease, and cancer

**DOI:** 10.1186/s12916-014-0250-y

**Published:** 2015-01-16

**Authors:** Massimo Franchini, Giuseppe Lippi

**Affiliations:** Department of Hematology and Transfusion Medicine, C. Poma Hospital, Strada Lago Pajolo 10, 46100 Mantova, Italy; Laboratory of Clinical Chemistry and Hematology, Academic Hospital of Parma, Via Gramsci 14, 43100 Parma, Italy

**Keywords:** ABO blood group, Cancer, Cardiovascular disease, Mortality, von Willebrand factor

## Abstract

Other than being present at the surface of red blood cells, the antigens of the ABO blood group system are efficiently expressed by a variety of human cells and tissues. Several studies recently described the involvement of the ABO blood group in the pathogenesis of many human disorders, including cardiovascular disease and cancer, so that its clinical significance extends now beyond the traditional boundaries of transfusion medicine. In a large cohort study recently published in *BMC Medicine* and including over 50,000 subjects, Etemadi and colleagues reported that nearly 6% of total deaths and as many as 9% of cardiovascular deaths could be attributed to having non-O blood groups, a condition that was also found to be associated with increased risk of gastric cancer. In this commentary, the clinical implications of ABO blood groups are critically discussed and a possible common pathogenic mechanism involving the von Willebrand factor is described.

Please see related article http://dx.doi.org/10.1186/s12916-014-0237-8.

## Background

**Figure 1:**
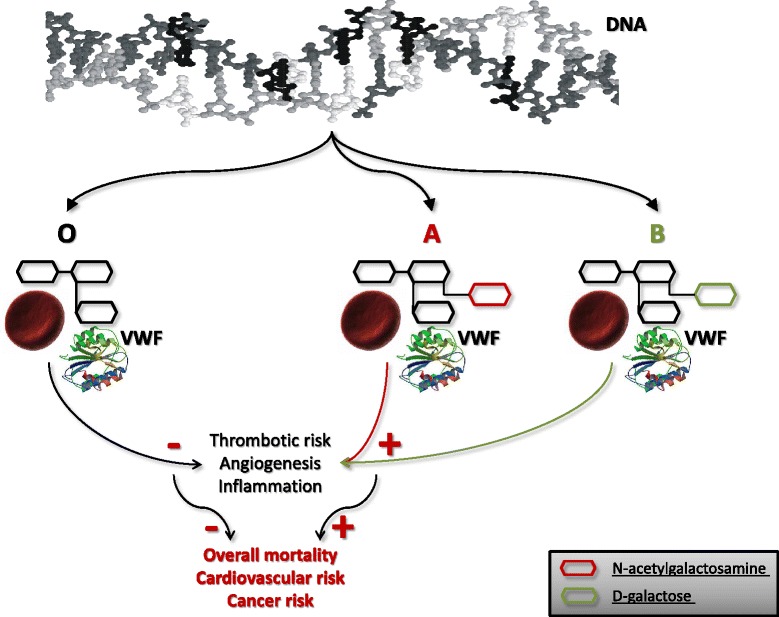
**The intriguing relationship between ABO blood group system, von Willebrand factor (VWF), cancer and cardiovascular disease.**

The antigens of the ABO blood group system (i.e., A, B, and H antigens), discovered more than one century ago [1], are complex carbohydrate molecules expressed on the extracellular surface of red blood cell membranes [2]. The A and B alleles encode slightly different glycosyltransferases that add N-acetylgalactosamine and D-galactose to a common precursor side chain, the H determinant, which is then converted into A- or B-antigens, respectively (Figure 1). The O alleles do not encode a functional enzyme, so that OO carriers lack these transferase enzymes and express the unaltered H structure, with a solitary terminal fucose moiety attached to the precursor oligosaccharide chain, which represents the phenotypic marker of the O blood group [3]. In addition to the expression on red blood cell surfaces, the ABO antigens are also present in a variety of human cells and tissues, including epithelium, sensory neurons, platelets, and vascular endothelium [4,5]. Therefore, it is not surprising that the clinical significance of the ABO blood group extends now beyond the traditional boundaries of immunohematology and transfusion medicine, wherein this antigen system is seemingly involved in the pathophysiology of a wide range of human diseases, the most important being represented by cancers and infectious and cardiovascular disorders [6-9].

## Discussion

A number of studies conducted in the past 50 years have consistently described the existence of an association between ABO blood type and cardiovascular disease [10-12]. In particular, a recent systematic review and meta-analysis documented that having a non-O blood group carries an approximately two-fold increased risk of venous thrombosis [10]. A weaker but still significant association was found in another systematic review conducted by the same group of authors between non-O blood type and arterial thrombosis (odds ratio [OR] of 1.28 for myocardial infarction and 1.17 for ischemic stroke) [11]. In addition to the effect of the ABO blood group on low-density lipoprotein and total serum cholesterol levels [13], the leading underlying mechanism that has been put forward to explain this association involves the profound influence that the ABO blood group system exerts on hemostasis, particularly on the von Willebrand factor (VWF) and, consequently, on coagulation factor VIII (FVIII) plasma levels, which are both well recognized prothrombotic risk factors [14]. Indeed, it is now clearly acknowledged that individuals of non-O blood group status have plasma levels of both VWF and FVIII that are approximately 25% higher than O blood group subjects [15]. The molecular basis of this phenomenon has been precisely identified with the presence of ABH antigenic structures on circulating VWF, which modulate the activity of this multifunctional protein through different degrees of glycosylation [12].

Another interesting field that has been extensively studied over the past five decades is that of the association between ABO blood group types and cancer [8,9]. The most consistent association has been found with pancreatic and gastric cancers [8]. For instance, in the Nurses’ Health Study and Health Professionals Follow-up Study, Wolpin et al. [16] found that participants with blood groups A, AB, or B were more likely to develop pancreatic cancer compared with those with blood group O (adjusted hazard ratio [HR]: 1.44; 95% CI: 1.14–1.82). The higher prevalence of blood group A in patients with gastric cancer formerly observed by several studies [9] has also been recently confirmed in a large prospective population-based study involving more than one million of Scandinavian blood donors followed for up to 35 years [17]. The strength of this association was similar to that previously reported (OR: 1.20; 95% CI: 1.02–1.42). Although the underlying mechanisms linking the ABO blood group system and cancer are still largely unknown, one plausible explanation involves the ABO blood group-driven regulation of circulating levels of several proinflammatory and adhesion molecules (i.e., soluble E-selectin, P-selectin, and intercellular adhesion molecule-1), which play a key role in the tumorigenesis process [9]. Moreover, the recent discovery that VWF is an important modulator of angiogenesis and apoptosis provides an alternative, particularly intriguing, hypothesis to unify the mechanisms by which non-O blood group influences the onset of cardiovascular and neoplastic diseases (Figure 1) [18]. A significant advance in this field has now been provided by the Golestan Cohort Study, recently published in *BMC Medicine* [19]. This large epidemiological trial analyzed the association between ABO blood groups and overall and cause-specific mortality in over 50,000 people recruited between 2004 and 2008. Notably, the authors found that non-O blood groups were associated with a significantly increased risk of total death (HR: 1.09; 95% CI: 1.01–1.17) and mortality for cardiovascular disease (HR: 1.15; 95% CI: 1.03–1.27). Although no significant association was found with ABO-related cancer mortality, an aspect that was investigated for the first time in this study, an increased risk of developing gastric cancers was still observed in individuals with blood groups A and B. This latter finding is particular intriguing, and is also in keeping with the results from another recent study conducted by our group, in which a negative association between B blood group and life expectancy in a large cohort (n = 28,129) of subjects was found [20]. Although the analysis was only limited to overall mortality in our study, this evidence may be attributable to the association between B blood type and some aging associated conditions, including neurological and neoplastic disorders. Although additional research is needed to corroborate these preliminary findings, the attractive data that have emerged from these studies raise a new and intriguing scenario linking the ABO blood group with cardiovascular disease and cancer (Figure 1).

## Conclusions

Despite being studied for more than half a century, the complex interplay between the ABO blood group system and human health is far from being definitely elucidated. In particular, if the association between non-O blood type and cardiovascular disease mortality is confirmed by further trials like that recently published by Etemadi et al. [19], non-O blood group status may be included in cardiovascular risk scores to better estimate the individual thrombotic risk profile. Further experimental studies are also needed to unravel the molecular mechanisms linking ABO blood type, VWF, and cancer development. Intuitively appealing, ABO blood typing may hence become part of a multifaceted strategy for cancer risk assessment.

## Abbreviations

FVIII: Coagulation factor VIII
HR: Hazard ratio
OR: Odds ratio
VWF: von Willebrand factor
